# Back pain and body posture of non-professional Brazilian Jiu-Jitsu practitioners

**DOI:** 10.7717/peerj.12838

**Published:** 2022-03-03

**Authors:** Katarzyna Sędek, Aleksandra Truszczyńska-Baszak, Anna Katarzyna Cygańska, Justyna Drzał-Grabiec

**Affiliations:** 1 Faculty of Rehabilitation, Józef Piłsudski University of Physical Education in Warsaw, Warsaw, Poland; 2 Medical Faculty, Institute of Physiotherapy, University of Rzeszow, Rzeszów, Poland

**Keywords:** Martial arts, Body posture, Back pain, Jujitsu, Preventive measures

## Abstract

**Background:**

The aim of the study was to assess the prevalence of back pain in non-professional Brazilian Jiu-Jitsu practitioners (NP-BJJ practitioners), and to assess the relationship between their back pain and postural disorders.

**Methods:**

The study involved 61 subjects (age: 30.7 ± 4.9 years old; body mass: 79.4 ± 12.9 kg; body height: 179.6 ± 8.4 cm; 8 woman and 53 man), 31 who trained Brazilian Jiu-Jitsu (BJJ) and 30 subjects who had never trained any combat sport. The mean time of training BJJ in the study population was 3.9 ± 4.1 years. Postural assessments were conducted with the use of the photogrammetric method. The assessment of back pain and injuries was conducted with the Oswestry Disability Index (ODI) and with our proprietary questionnaire.

**Results:**

Among both populations, 37 subjects reported back pain. There were no differences in back pain location between the two groups or in functional state on the ODI. Significant differences between the groups in the values of the postural parameters (spinal height, length of kyphosis, length of lordosis, pelvis inclination angle) were observed. Statistically significant differences between the NP-BJJ practitioners with and without back pain in the length of the thoracic kyphosis and the differences in the height of the waist triangles were found.

**Conclusions:**

Both in the study population and in the control group the spinal pain was minimal or moderate on the ODI. BJJ practitioners who reported back pain had characteristic postural changes in some of the analysed postural parameters in comparison to BJJ practitioners who did not report back pain.

## Background

The development of the sports discipline of Brazilian Jiu-Jitsu (BJJ) and its increasing popularity was followed by an increasing number of publications on the physical and physiological profile of BJJ athletes (Andreato et al., 2017), on the biomechanics aspects of the athletes (de Paula Lima et al., 2017), on the technical and tactical analyses (Andreato et al., 2015; Andreato et al., 2013), and on the incidence of injuries (Spano et al., 2019; Das Graças et al., 2017; Scoggin et al., 2014; Kreiswirth, Myer & Rauh, 2014). BJJ is a type of combat sport and as such it requires certain physical and physiological qualities, such as good muscle development, aerobic and anaerobic power, muscle power and endurance and flexibility (Andreato et al., 2017; de Paula Lima et al., 2017). Studies on prevalence of injuries in BJJ found that its athletes, similarly to those who do other types of combat sport, suffer from frequent injuries. Depending on the study, as many as 25.5% (Spano et al., 2019) to 85.7% (McDonald et al., 2017) athletes reported having had an injury. The most common were orthopaedic injuries (78%) (Scoggin et al., 2014). The injuries affect the limbs as well as the torso (McDonald et al., 2017; Moriarty, Charnoff & Felix, 2019a; Jensen et al., 2017; Wisdom & Valleser, 2016), and internal organs (kidneys) (Itagaki & Knight, 2004). It has been found that athletes sustain more injuries during training than during competitions (Petrisor et al., 2019).

Apart from injuries sustained during training different sport disciplines and competition, athletes suffer from back pain (Yamashita et al., 2019; Sairyo & Nagamachi, 2016; De Luigi, 2014; Daniels et al., 2011), usually in the lumbar spine and a lifetime prevalence reaches up to 94% (Trompeter, Fett & Platen, 2017). Depending on the test and the time when examinations were conducted (preparation, competition, interim) the point prevalence ranging from 18 to 65% (Trompeter, Fett & Platen, 2017). The only report available in the literature which discusses the issue of back pain in BBJ athletes is from Reis et al. (2015). They found that BJJ athletes of different levels of advancement experience both acute and chronic back pain. Therefore, the natural direction of examination in searching for causes and in assessment of an athlete with back pain is the analysis of their posture. Several authors have analysed posture of athletes of different sports disciplines (Özyürek, Bayraktar & Genç, 2018; Walaszek et al., 2017; Grabara, 2020; Kruusamäe et al., 2015; Pereira et al., 2019). However, no report on BJJ postural assessment had been found.

Most papers available from the literature on BJJ discuss professional athletes (Spano et al., 2019; Das Graças et al., 2017). In the literature only two papers were available, which focused on the athletes who did BJJ both non-professionally and/or recreationally (Wisdom & Valleser, 2016; Reis et al., 2015). The population of non-professional BJJ athletes is as big as the population of professional BJJ athletes. Moreover, a professional athlete is supported by qualified training staff, has access to sports base and medical care; while non-professional athletes or persons doing recreational training cannot benefit from that type of support.

The aim of the study was to assess the prevalence of back pain and body posture in non-professional Brazilian Jiu-Jitsu practitioners in comparison to control group, and to assess the relationship between their back pain and postural disorders.

## Methods

### Participants

The study is observational and involved 61 subjects. The study population consisted of 31 non-professional Brazilian Jiu-Jitsu practitioners, recruited from sports clubs. The control group were 30 subjects-recreational sport practitioners who had never done combat sport. The participants constituted a convenience sample. Each participant was informed about the purpose and procedure of the research, as well as the of possibility of withdrawing at any time during the study. All participants gave their written, informed consent to participate in the project. All experimental protocols were approved. Authors obtained the consent to conduct the study from the Senate Ethics Committee for Scientific Research (SKE 01-45/2016) and confirmed conducting research to the standards set by the Declaration of Helsinki.

**Figure 1:**
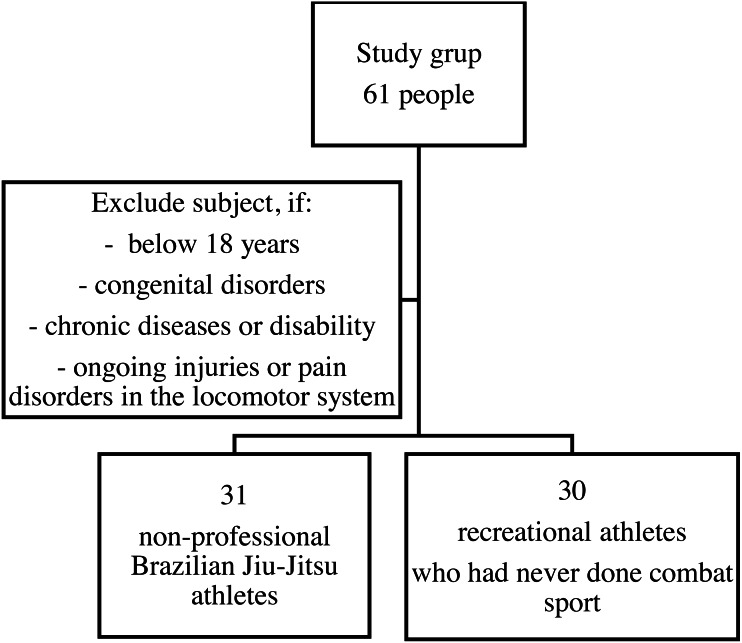
A sample selection flowchart.

The criteria for subject inclusion in the study population were: subject both sexes, age between 18 and 45 years old, having trained BJJ for a minimum of 6 months with the duration of a minimum of 1.5 hours per week, having trained BJJ non-professionally (without participation in competitions and contests). The criteria for subject inclusion in the control group were: age between 18 and 45 years old, different level, being physically active except for training BJJ or any other sports professionally. A sample selection is presented on a flow chart Fig. 1.

The subjects’ age was between 24 and 43 years old (in the study population it was 31.7 ± 5.2 years old, and in the control group it was 29.7 ± 4.4 years old). In each group was 4 women. We did not find statistically significant differences between body mass or body height between the two groups. Table 1 presents the biometric data of the study population subjects.

The mean time of training BJJ in the study population was 3.9 ± 4.1 years. Amount of subject depends on training time presents Table 2. The BJJ practitioners trained a mean of 4.06 ± 2.8 times per week, and the training sessions were 1.5 hours long. Unfortunately, it was not possible divide the BJJ practitioners regarding the fighting style (pass fighters and guard fighters) or an intragroup sub-analysis by practice time because of small number of subjects in particular subgroups.

### Postural stability assessment

Postural stability assessment was conducted with the use of the photogrammetric method based on the projection of the moiré method. The equipment for the computer-based body posture assessment system Mora 4th Generation was used. This method is non-invasive, easy to use, easily available, and it is easy to analyse the obtained results. Moreover, the photogrammetric method allows for comparison of results, as it is increasingly often used in postural assessment studies. All the measurements were taken by the same researcher, a physiotherapist (K.S.), trained to operate the equipment while maintaining the constant conditions and procedure of the tests. Before the measurements were conducted a suitable room was adapted for the tests. The tests need to be conducted in a darkened room, so the windows were curtained, and the space in the room was organized properly so as to ensure safe and non-embarrassing conditions for conducting the measurements (the subjects needed to undress for the tests). To calibrate the Mora4G correctly, before each measurement session the device was adjusted using the spirit levels on the front and the side of the device. The researcher measured subjects’ body mass and body height, and then, with the subject standing, marked palpated bony landmarks with a marker pen (spinous processes C7-S1, cervico-thoracic junction, thoracolumbar junction, scapula inferior angles, spina iliaca posterior superior, kyphosis peak, lordosis peak). To ensure comfortable and non-embarrassing conditions, female subjects could use a specially prepared cloth to cover the chest, attached to the body with adhesive tape. Subjects stood with their back to the projector-receiver device at a constant distance of 2.6 m, with their feet placed under their hips and hands along the body, eyes looking forward. Then, the measurement was taken. According to the producer, the measurement error with the MORA 4G system is one cm at a maximum (CQ Elektronik System, 2018). To analyse the photogrammetric measurements, we used the following parameters were used:

**Table 1:** Subjects biometric data.

Descriptive statistics	Age (years)		Body mass (kg)		Body height (cm)		BMI (kg/m)	
	$\bar {x}$	SD	$\bar {x}$	SD	$\bar {x}$	SD	$\bar {x}$	SD
Study population (*n* = 31)	37.74	5.22	81.16	10.44	179.06	7.73	25.3	2.83
Control group (*n* = 30)	29.67	4.36	77.53	14.93	180.2	9.15	23.74	3.24
Total (*n* = 61)	30.72	4.89	79.38	12.87	179.62	8.41	24.53	3.11
Statistical significance (*p*)	*Z* = 1.52 *p* = 0.126		*Z* = 1.31 *p* = 0.191		*Z* = − 0.62 *p* = 0.535		*Z* = 2.19 *p* = 0.029	
Effect size	0.038		0.028		0.006		0.079	

**Table:** 

DCK (%)	Spinal height from C1 to S1
ALFA (°)	Lumbosacral region inclination
BETA (°)	Thoracolumbar region inclination
GAMMA (°)	Upper thoracic region inclination
DKP (mm)	The length of kyphosis between C7 and peak of kyphosis
KKP (°)	Thoracic kyphosis angle
RKP (mm)	The length of kyphosis between C7 and thoracolumbar junction
GKP (mm)	The depth of thoracic kyphosis
DLL (mm)	The length between S1 and peak of lordosis
KLL (°)	Lumbar lordosis angle
RLL (mm)	The length of lordosis between S1 and thoracolumbar junction
GLL (mm)	The depth of lumbar lordosis
KLB (°)	Shoulder line inclination difference
UB (°)	The difference in the depth of scapula inferior angles inclination
OL (%)	The difference between the distance of the scapula inferior angles from the spine
TT (%)	Difference in the height of the waist triangles
TS (%)	Difference in the width of the waist triangles
KNM (°)	Angle of pelvis inclination
KSM (°)	Angle of pelvis rotation

### Questionnaire

For the purpose of this study, the Polish validated version of the Oswestry Disability Index (ODI) was used (Miekisiak et al., 2013). The ODI is used for the functional state assessment of patients with low back pain and it is the golden standard for the functional assessment in this group of patients. The questionnaire is accessible, patients can complete it on their own and it can be completed in a few minutes. The subjects also filled out the author’s original questionnaire consisting of 8 questions which comprised of the following: the biometric data (age, body mass, sex), data on the subjects’ physical activity (duration, intensity, type), and injuries subjects sustained as well as spinal pain (location, symptoms, causes, pain intensity etc.).

**Table 2:** Amount of subject depends on training time (years).

Training time (years)	Total (*n*)
<2	13
2–3	9
4–5	2
<5	7

### Statistical analysis

Statistical analysis of the collected data was conducted with the STATISTICA 13 programme (TIBCO Software Inc., Palo Alto, CA, USA). Quantitative data was presented in the form of descriptive statistics using such measurements as arithmetic mean and standard deviation. The relationships between qualitative data were assessed with the chi-squared test, and the differences in two groups in quantitative data were assessed with the Mann-Whitney *U*-test. The non-parametric test was chosen as there was lack of normal distribution in the analysed variables. Normal distribution was verified with the Shapiro-Wilk test. Statistical significance was set at *p* < 0.05. Effect sizes were computed using method proposed by Tomczak & Tomczak (2014).

## Results

### Back pain and analysis of sustained injuries

Current back pain was present in 37 subjects (60.7%), including 22 (71.0%) BJJ practitioners and 15 (50.0%) controls (*p* = 0.094). The pain usually affected the lumbar spine (44.3%), and it was less common in cervical spine (13.1%) and thoracic spine (18.0%). There were no differences in pain topography between the two groups, *p* >0.05. Pain intensity on the Numeric Rating Scale, NRS (from “0” meaning no pain to “10” meaning pain as bad as you can imagine) was 4.09 ± 1.95 points in the study population and 3.4 ± 1.59 point in the control group and it was statistically insignificant, *p* = 0.3.

The subjects’ disability was between minimal and moderate as shown by the ODI, and their results did not show statistically significant differences (215% for the studied population, *n* = 22; 20% for the control group, *n* = 15).

The most commonly reported injuries by study population subjects sustained because of BJJ training affected the knee (32.3%), the auricle and the ankle (29.0% each), and the elbow (16.1%).

There were no statistically significant differences between the groups in physical activity (*p* = 0.654). In the study population, 28 subjects (90.3%) declared physical activity beside BJJ, with 26 subjects (86.7%) in the control group. Training of other sports (beside BJJ) was done by subjects from both groups. BJJ practitioners had other physical activity (beside BJJ) statistically significantly less often than the controls (*p* = 0.027). The study population did it at a mean of 3.89 ± 3.16 times per week, and the control group 5.73 ± 3.47 times per week. Other forms of physical activity of subjects from both groups included power workout (50.8%), stretching (32.8%), swimming (29.5%), running (24.6%) and cycling (19.7%). Subject from control group went swimming statistically significantly more often than the study population subjects (*p* = 0.004).

### Body posture analysis

We observed statistical differences between the two groups in the values of four of the studied parameters: DCK- spinal height, *p* = 0.001, RKP- kyphosis height calculated between C7 and thoracolumbar junction, *p* = 0.010, RLL- lordosis height calculated from S1 to the thoracolumbar junction, *p* = 0.009 and KNM- angle of pelvis inclination, *p* = 0.041. For each of these four parameters, the study population subjects had higher values than controls. Moreover, the BETA parameter—thoracolumbar region angle and the TT parameter—difference in the height of the waist triangles had lower values in the study population. These values were close to the statistical significance, *p* = 0.063 and *p* = 0.056, respectively. Table 3. presents studied parameters value.

**Table 3:** Photogrammetric measurement results in the study population and in the control group.

Analysed parameters	Study population (*n* = 31)			Control group (*n* = 30)			Z	*p*-Value	Effect size
	$\bar {x}$	Me	SD	$\bar {x}$	Me	SD			
DCK (%)	474.8	473.5	25.4	447.1	448.0	25.9	3.73	0.001^*^	0.228
ALPHA (°)	5.04	3.80	3.60	4.19	3.85	2.22	0.34	0.735	0.002
BETA (°)	7.09	6.90	1.97	8.29	7.85	2.26	−1.86	0.063	0.057
GAMMA (°)	14.14	10.70	11.28	14.30	10.10	12.90	1.10	0.273	0.020
DKP (mm)	188.0	190.9	21.0	194.4	189.5	22.6	−1.11	0.267	0.020
KKP (°)	158.8	162.2	11.4	157.4	161.6	13.4	0.27	0.790	0.001
RKP (mm)	321.9	327.0	15.2	307.3	307.7	22.2	2.59	0.010^*^	0.110
GKP (mm)	16.58	16.60	6.58	16.43	15.45	5.85	0.02	0.983	0.000
DLL (mm)	95.1	98.3	20.9	87.4	87.5	19.6	1.51	0.132	0.037
KLL (°)	168.8	169.2	5.2	168.1	168.2	4.2	1.00	0.320	0.016
RLL (mm)	152.9	153.1	18.2	139.8	135.2	17.6	2.60	0.009^*^	0.111
GLL (mm)	7.14	5.30	5.53	7.18	7.15	3.53	−0.85	0.395	0.012
KLB (°)	8.69	6.60	7.68	8.00	6.60	6.52	0.19	0.851	0.001
UB (°)	5.25	4.50	4.52	4.72	4.50	3.69	0.17	0.868	0.000
OL (%)	11.81	10.90	9.03	8.96	6.65	7.01	1.46	0.143	0.035
TT (%)	9.04	8.60	7.04	13.11	10.95	8.38	−1.91	0.056	0.060
TS (%)	8.05	7.60	5.43	7.91	7.60	6.92	0.47	0.639	0.004
KNM (°)	2.24	2.40	1.18	1.64	1.30	1.34	2.05	0.041^*^	0.069

**Notes.**

Me median Min minimum Max maximum SD standard deviation *Z* result of the Mann–Whitney *U* test *p*-Value level of significance of differences

* *p* < 0.05.

The relationship between back pain and postural disorders assessed as a difference in body posture in BJJ practitioners with and without back pain and comparison their photogrammetry parameters with control group. Statistically significant differences between subjects training BJJ with and without back pain from the study population in the RKP - the length of the thoracic kyphosis were found. The RKP parameter values (BJJ practitioners with pain 317.7 ± 14.7 vs. BJJ practitioners without pain 332.2 ± 11.7) were statistically significantly lower in subjects with pain (*p* = 0.01; effect size = 0.110). Statistically significant differences between subjects training BJJ shorter than 3 years (*n* = 18) and longer/or 3 years (*n* = 13) from the study population in the KKP—thoracic kyphosis angle and in the gamma angle - upper thoracic region inclination. The KKP parameter values (BJJ <3 years 162.74 ± 2.88 vs. BJJ ≥3 years 153.28 ± 16) were statistically significantly lower in subjects longer training internship (*p* = 0.02; effect size = 0.18). The gamma angle values (BJJ <3 years 10.21 ± 2.73 vs. BJJ ≥3 years 19.58 ± 15.89) were statistically significantly lower in subjects longer training internship (*p* = 0.02; effect size = 0.17). The mean training time in BJJ practitioners with back pain was 3.5 years, and in BJJ practitioners without back pain 4.7 years. There were no statistically significant differences in values of the studied parameters among the control group subjects with and without pain. The multivariate discriminant analysis confirmed above results (Canonical correlation = 0.731; Wilks’ Lambda = 0.465; *p* = 0.002). Data does not support the thesis on protective influence of BJJ practice. Odds Ratio computed for actual back pain experience is 1.491, CI _.95_ (.93−2.16), so it is only close to significance, for BJJ practitioners. Our study remarks the subclinical effects, detectable in photogrammetric analyses.

## Discussion

In this study, both people training BJJ and people from the control group declared the occurrence of back pain. Pain frequency, location (in both groups it usually affected lumbar spine) and intensity were similar in both groups (between minimal and moderate on the ODI). It would be recommended for these subjects to receive suitable instructions on pain management in activities of daily life, *i.e.*, on lifting objects, sitting or performing certain physical exercise correctly (Miekisiak et al., 2013). In the study by Reis et al. (2015) 35.6% recreational athletes (*n* = 36) reported LBP, the results of the Quebec Back Pain Disability Scale (QBPDS) was low in both populations (professionals, Me:10; recreational, Me:6). Authors showed that professionals had a marginal increase in the risk factor of developing LBP in comparison to recreational sportsmen.

Sedentary lifestyle facilitates development of LBP. Although optimal dose of exercise and physical activity has not been determined (Heneweer et al., 2011), it seems justified to introduce modifications and adequate adaptation of BJJ training in recreational BJJ athletes with LBP. It seems important that subjects with back pain participate in training in the afternoon or evening instead of morning hours (Moriarty, Charnoff & Felix, 2019a). Moreover, athletes assume numerous position during training, particularly in numerous guards, and crossing the guard of the other contestant is an element of the bout (de Paula Lima et al., 2017). These activities may generate loads for the spine because of alternating from maximal flexion to maximal extension. A recommendation for athletes with LBP may be using half-guard in training, so that the opponent does not lay his whole mass on the spine of the guard-keeping athlete. This would definitely reduce loads to the lumbar spine (Moriarty, Charnoff & Felix, 2019a). Sung-jun, Kyue-nam & Moon-soo (2018) observed changes to hip rotation in BJJ athletes related to occurrence of LBP. This suggests the necessity for athlete assessment with regard to disorders and impairments of the locomotor system (movement range in the joints, muscle strength, movement system impairments) prior to training, or prior to those training elements which particularly load the lower spine.

Studies on injuries in BJJ found that only 6% of injuries happen during contests (Petrisor et al., 2019) and that these are usually orthopaedic injuries (Scoggin et al., 2014) affecting mainly knees and elbows (Kreiswirth, Myer & Rauh, 2014). Most injuries happen during training (Petrisor et al., 2019) and they usually affect distal limb parts, *i.e.*, hands, feet, fingers and toes (McDonald et al., 2017). Injuries which are reported to doctors or physical therapists usually affect lower extremities, while injuries to upper extremities, more commonly sustained during training, are probably not serious enough to require medical consultation (McDonald et al., 2017; Wisdom & Valleser, 2016). Injuries to the back (14.6%) are the third most common type of injury, after injuries to the lower extremities (38.1%) and to the upper extremities (32.3%). Here, the subjects usually reported injuries to the cervical spine (Moriarty, Charnoff & Felix, 2019b). In authors own study, the most commonly reported injuries sustained because of BJJ training by study population subjects affected the knee (32.3%), the auricle and the ankle (29.0% each), and the elbow (16.1%). Injuries are more common in professional athletes and, as studies have shown, happen mainly in contests or during immediate preparation for contests. This may explain the smaller number of serious injuries in recreational athletes. The injuries they sustain are less serious than in professional athletes (Wisdom & Valleser, 2016).

In authors own study changes to BJJ athletes’ body postures in comparison to inactive subjects were found. These changes were found in four of the studied parameters (DCK, RKP, RLL and KNM). Statistically significant differences between subjects with and without back pain from the study population in the RKP parameters were found. BJJ athletes with LBP had shorter training time than BJJ athletes without LBT, which might suggest that BJJ has positive impact on body posture and LBP incidence. Similarly, to Walaszek et al. (2017) found that regular 6-month judo practice had positive effect on young judo athletes’ body posture, and Grabara (2020) found that two years of volleyball training did not result in asymmetries in teenage athletes posture. de Queiroz et al. (2016) found improvement of functional fitness, health and quality of life in elderly men who trained BJJ for 12 weeks. Postural disorders in athletes may result from the specificity of the sports discipline (repeated movements, asymmetric movements, wrong technique) (Piwowarski, 2016), yet they may also be related to the past injuries. Regular recreational physical activity, *e.g.*, BJJ may result in greater wellbeing and positive changes on body posture.

## Limitations

The Mora system has numerous advantages, yet there is still certain risk of measurement error involved. Authors have tried to minimize the error in that all the measurements were conducted by the same researcher. Another limitation is that the intra-rater correlation index has not been calculated. Unfortunately, no normative values are available for the Mora 4G that would allow us to refer the obtained results to them, and thus increase the value of the conducted analysis. Follow-up studies should include back pain diagnosis based on clinical examinations and possibly medical imaging. They should also focus on reasons why certain athletes have declined to participate in the study, or reason for absence of some athletes in training sessions (possibly related to back pain or an injury). This would allow for adjusting training and prevention for this sports discipline. The number of participants is also a limitation. As the study population was small pain reported or postural differences separately for men and for women were not analysed. The study design could be extended to include additional measures and extended statistical analyses. The above-mentioned limitations do not decrease the value of this paper by any means. They may be treated as indications for further studies. Accepting these limitations, this article highlights differences in body postures in non-professional BJJ practitioners in comparison to subjects who do not train this discipline. This is the first study to report postural changes in BJJ practitioners reporting back pain in comparison to BJJ practitioners without pain. This is also the first study to report how common lumbar spine pain is in recreational BJJ practitioners.

## Conclusions

1. There are no significance differences in back pain in regards to its frequency, intensity and location between non-professional BJJ practitioners and subjects who do not train BJJ.
2. Both in the study population and in the control group the lower back pain was minimal or moderate on the Oswestry Disability Index.
3. BJJ practitioners who reported back pain had characteristic postural changes in some of the analysed postural parameters in comparison to BJJ practitioners who did not report back pain.

## Supplemental Information

10.7717/peerj.12838/supp-1 Supplemental Information 1 Raw data The measurements for all participants in two separated groups.

10.7717/peerj.12838/supp-2 Supplemental Information 2 The author’s original questionnaire consisting of 8 questions

## Additional Information and Declarations

### Data Availability

The following information was supplied regarding data availability: The raw measurements are available in the Supplementary File.
