# Supplementary material for: Back pain and body posture of non-professional Brazilian Jiu-Jitsu practitioners
Source: PeerJ. 2022 Mar 3;10:e12838. doi: 10.7717/peerj.12838 (PMC8898545; doi:10.7717/peerj.12838)
Supplement: Supplemental Information 2 [file peerj-10-12838-s002.docx]

**Survey questionnaire**

**Dear Participants,**

I am a student of Rehabilitation Department of the Warsaw Academy of Physical Education.
I am writing a master’s thesis to analyze the impact of Brazilian jiu-jitsu on preservation of the natural spine curves and spine pain.

I am asking you to complete the following survey because it will help me gather materials for my work.

Best regards and thank you for your time,

Katarzyna Sędek

Survey Questions:

1. Name and surname

………………………………………………………………………………………..

1. Body weight: ………….. kg
2. Body height: ………….. cm
3. How long have you been training Brazilian jiu-jitsu? (if applicable)

………………………………………………………………………………………..

1. How many times a week do you train? (if applicable)
2. Do you engage in other physical actvities? How many times a week?

□ Swimming x…………

□ Running x…………

□ Cycling x…………

□ Stretching x…………

□ Strength training x…………

Other. What activities? ……………………………………………………………………………………………….……….…….............………………………………………………………………………...

1. Do you have pain in the cervical/thoracic/lumbar spine? (If yes, underline approperiate part of the spine)

□ No.

□ Yes.

To what extent?


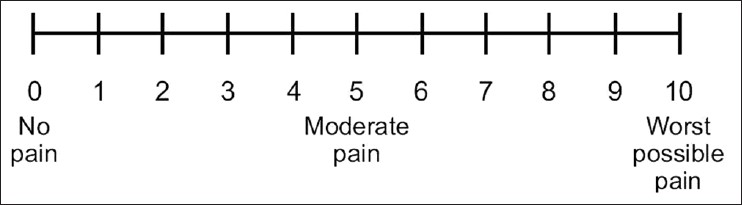


1. Have you suffered any other injuries?

□ No.

□ Yes.

What injuries?

|  | Please enter the area of the body affected by the injury. | Was the injury related to Brazilian jiu-jitsu? [YES/NO] |
| --- | --- | --- |
| Bone fracture |  |  |
| Joint dislocation |  |  |
| Muscle damage |  |  |
| Ligament damage |  |  |
| Skin break |  |  |
| Auricle damage | X |  |
